# The role of point-of-care ultrasound in the diagnosis and management of necrotizing soft tissue infections

**DOI:** 10.1186/s13089-020-0153-4

**Published:** 2020-01-23

**Authors:** Luís Magalhães, Sara Raquel Pereira Martins, Ramon Nogué

**Affiliations:** 1 Hospital da Luz–Arrábida, Praceta de Henrique Moreira 150, 4400-346 Vila Nova de Gaia, Portugal; 2 Centro Hospitalar Universitário do Porto, Largo do Prof. Abel Salazar, 4099-001 Porto, Portugal; 3 Universitat de Lleida, Plaça de Víctor Siurana, 1, 25003 Lleida, Spain

**Keywords:** Soft tissue infections, Ultrasonography, Emergency Medicine, Point-of-care testing, Diagnostic imaging

## Abstract

**Background:**

Necrotizing soft tissue infections are associated with high morbidity and mortality, even when the correct treatment is initiated. The diagnosis of these conditions is hard and the most sensitive methods are time-consuming, expensive and not readily available. Point-of-care ultrasound can complement clinical evaluation to increase the diagnostic accuracy.

**Case presentation:**

We bring a case of a woman, without comorbidities, who presented to the emergency department with signs of soft tissue infection. Bedside ultrasound showed subcutaneous tissue thickening, with fluid accumulation, and subcutaneous gas in the affected area. Based on the clinical suspicion and the ultrasound findings, the patient underwent prompt medical treatment and surgical debridement.

**Conclusions:**

This case shows the utility of bedside ultrasound for the decision-making process in a disease where an early diagnosis is important. This information should always be used together with the clinical judgement, as it has a relative low sensitivity.

## Background

Necrotizing soft tissue infections (NSTI) are a group of infections characterized by a fulminant destruction and necrosis of the skin and soft tissue and that is frequently associated with systemic illness [1]. Even when the correct treatment is initiated, this condition is associated with a high mortality and morbidity, with mortality rates between 25 and 50% [2–5]. The diagnosis of these conditions is hard, with studies showing high rates of misdiagnosis at presentation [6], mainly because the initial clinical manifestations can be underestimated. An early institution of appropriate treatment, which includes early surgical intervention and antibiotic therapy, improves the outcomes. The diagnosis is primarily clinical, consisting of pain and swelling with minimum skin involvement that progresses to induration and formation of bullae. To complement the clinical evaluation, computerized tomography (CT) is the most used method of diagnosis. However, CT can have some drawbacks, namely being a time-consuming and expensive method that is not readily available in all clinical practice contexts. Magnetic resonance imaging (MRI) has a higher sensitivity and specificity, but have the same problems of CT. Given the severity and time-dependency of this condition, surgical intervention should not be delayed when the degree of suspicion is high.

Ultrasonography is a widely used method for evaluating the soft tissue. In recent years, point-of-care ultrasound (POCUS) has gained a lot of visibility between the medical community as a complement to clinical evaluation, as a fifth pillar of the physical examination [7]. Incorporating this technique can improve diagnostic speed and accuracy. Its use is well established in different areas, including the heart [8], lung [9], trauma [10], shock and even during cardiopulmonary resuscitation [11]. However, evaluation of the soft tissue performed by non-radiologists in the context of emergency is not so well established.

The purpose of this case is to demonstrate how POCUS can easily and quickly provide reliable additional information to the physical exam to support the diagnosis of NSTI and increase the confidence with which we stratify the severity of these patients and propose more invasive and expensive attitudes, such as CT or even surgical intervention.

## Case presentation

**Fig. 1:**
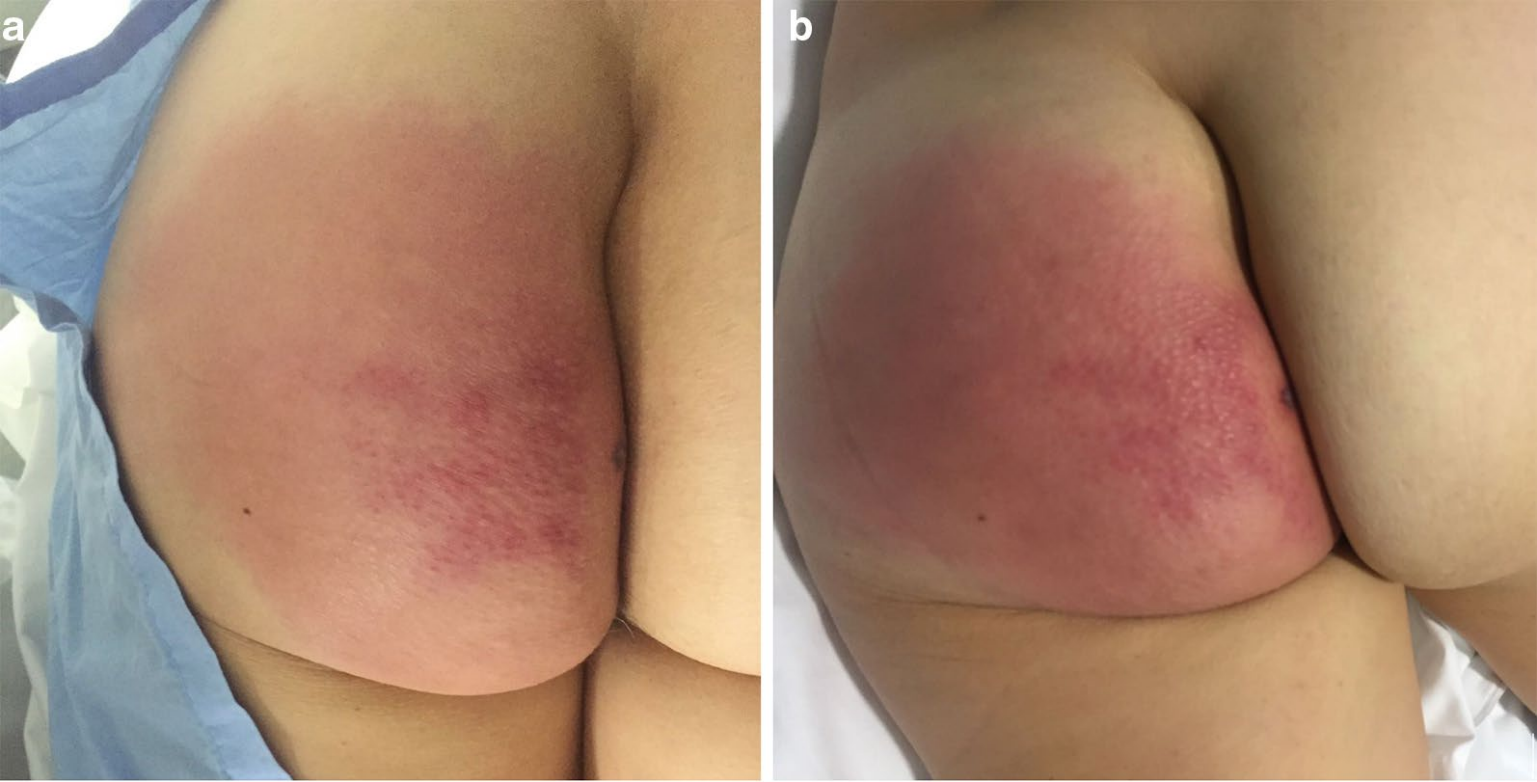
Macroscopic aspect of the left gluteus with signs of cutaneous infection: **a** at admission and **b** 3 h after admission, showing rapid progression of the redness around a central area of induration

We bring a case of a 54-year-old woman, without comorbidities, who presented to the emergency department with a 12-h redness and painful swelling in the left gluteal region. She denied trauma. At admission, she was afebrile (36.2 °C), with a normal blood pressure (109/65 mmHg), heart rate 89 bpm and mild peripheral oxygen desaturation (92% breathing room air), without other signs of respiratory distress. On the physical exam, she had a left gluteus induration with about 5 cm × 5 cm with redness in the surrounding area, as seen in Fig. 1a. There was no crepitus. The remaining physical exam was normal. Her initial labs were: hemoglobin 11.3 g/dL (*N* 13.5–17.5 g/dL), total white blood cell 6.550/μL (*N* 3.500–11.000/μL), 96% neutrophils (*N* 45–75%), platelets 164.000/μL (*N* 125.000–400.000/μL), creatinine 0.88 mg/dL (*N* 0.6–1.3 mg/dL), urea 51 mg/dL (*N* 22–52 mg/dL) and C-reactive protein 199.52 mg/dL (*N* < 5 mg/dL).

**Fig. 2:**
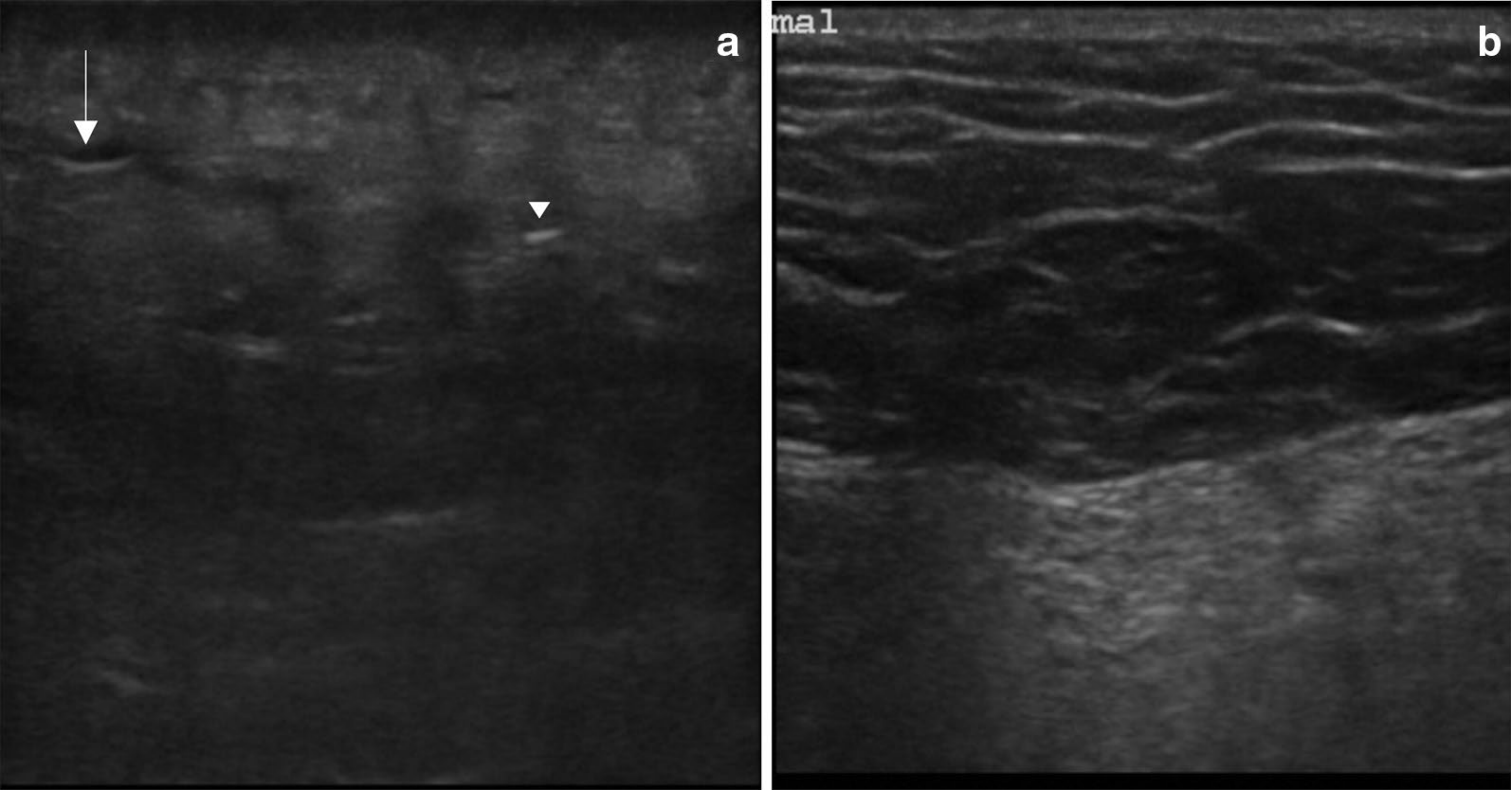
Point-of-care ultrasound scan of the affected left gluteal soft tissue (**a**) and normal soft tissue on the contralateral side (**b**) using a 15–6 MHz linear probe. In the affected tissue, there is loss of the normal subcutaneous architecture, diffuse increased echogenicity, hypoechoic regions that correspond to little fluid accumulations (arrow) and an hyperechoic focus with posterior dirty acoustic shadowing, corresponding to gas in the soft tissue (arrowhead)

A bedside ultrasound scan using a linear probe was performed which showed subcutaneous tissue thickening, with a “cobblestone” appearance, hypoechoic foci of fluid accumulation in the subcutaneous space, as well as the presence of gas in the tissue (Fig. 2a), significantly different from the normal skin (Fig. 2b).

**Fig. 3:**
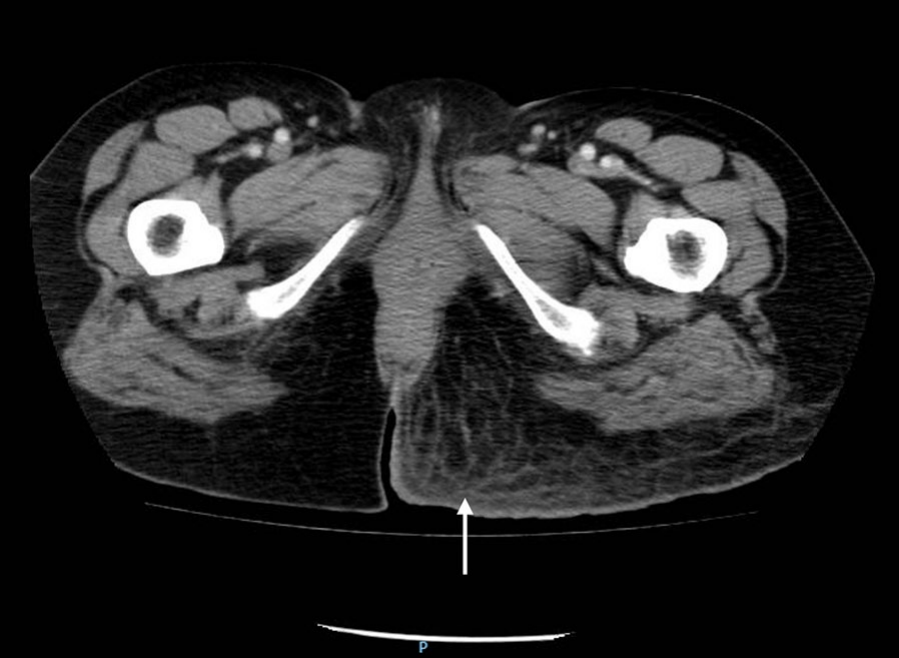
Contrast CT scan of the lower limbs on day 2 after surgical debridement. Axial tomogram at the level of the proximal thigh showing abnormal enhancement of the subcutaneous and fascial layer of the left gluteal region (arrow)

**Fig. 4:**
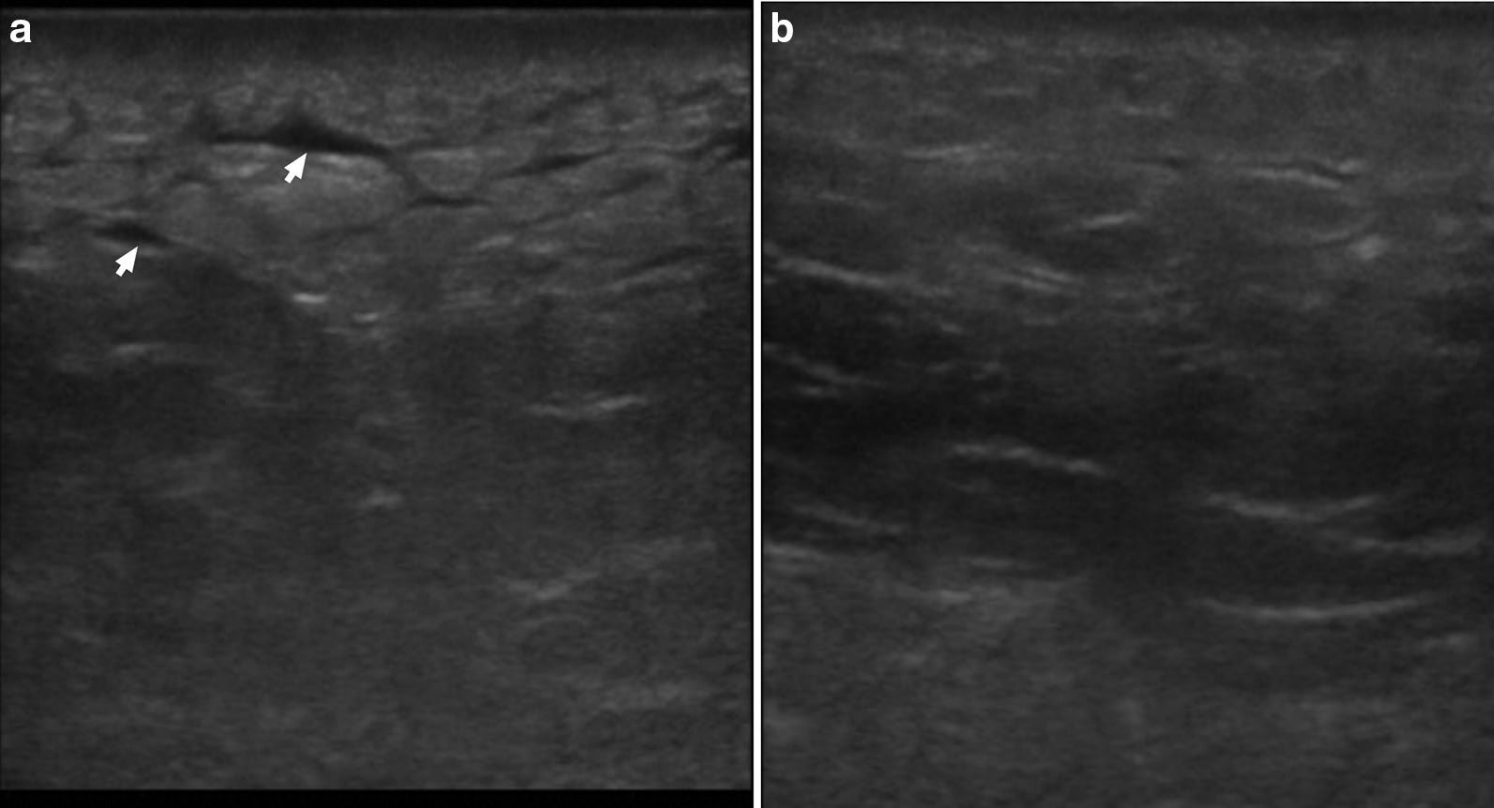
Point-of-care ultrasound scan of the affected left gluteal soft tissue 4 days after admission (**a**) and 8 days after admission (**b**) using a 15–6 MHz linear probe. These images show the progression of the sonographic appearance of NSTI, initially with accumulation of fluid in the subcutaneous tissue (arrows), giving it a “cobblestone” appearance and a progressive return to the normal architecture of the subcutaneous tissue over time

Even though the patient was initially stable, she was started on intravenous fluids and large spectrum antibiotics based on a high clinical suspicion for NSTI, supported with the ultrasound findings. After consultation with general surgery, considering the suggestive clinical and ultrasound findings, the presence of some alarming signs (tendentially low blood pressure, peripheral oxygen desaturation, elevated inflammatory parameters and rapid evolution of cutaneous inflammatory signs) and the time needed for the CT scan, unavailable at that moment, the patient underwent prompt surgical debridement to avoid delays in the treatment. In the procedure, the surgeon found necrotic tissue, purulent liquid and evidence of gas in the tissue, thus confirming the diagnosis. She was then transferred to the Intensive Care Unit, where, on the first day post-operative, evolved with septic shock, requiring vasopressor therapy. CT on day 2 showed no signs of additional complications (Fig. 3), namely enteric fistulas or bone involvement. She underwent one more major surgical debridement on day 3, as well as minor debridements and washouts, and was discharged 7 days later. Blood and tissue cultures were unable to identify the microorganism. Follow-up at 5 and 10 days after the discharge showed good evolution of the wound. The normalization of the image observed by ultrasonography gradually occurred after the fever, pain and the erythema was resolved (Fig. 4). The exam was normal at the 30-day post-discharge evaluation.

## Discussion

As mentioned, NSTI are a group of diseases with high mortality and morbidity, even when the correct diagnosis and treatment is made on time. Unfortunately, this is not an easy diagnosis, as the initial manifestations can be mistaken with more frequent and less severe diseases like cellulitis, erysipelas, diabetes mellitus decompensation, gastroenteritis and others [6]. More specific clinical signs, like large hemorrhagic bullae, skin necrosis, fluctuance, crepitus, and sensory and motor deficits are late signs of this condition. Clinical criteria have been developed to increase the accuracy of the clinical evaluation, namely the LRINEC Score [12], but the sensitivity and specificity are still not ideal [13]. Gas on plain radiographs was only seen in 16.9% in one series [14]. POCUS can improve diagnose accuracy for NSTI when used in combination with clinical evaluation as it is increasingly available, fast and can be performed at the bedside. One study estimated a sensitivity of 88.2% and specificity of 93.3% for the diagnosis on patients with clinically suspected NSTI [15]. The main findings of NSTI by ultrasound are reflected in this case and can be summarized in [15–18]: loss of the normal tissue architecture to a “cobblestone” appearance, with irregularity and thickening of the fascia, abnormal fluid collections along the fascia, seen as hypoechogenic zones, and, in more advanced cases, the presence subcutaneous air, defined by hyperechogenic foci with a posterior dirty acoustic shadowing. Even in the absence of crepitus at physical examination or plain radiographs, POCUS can show evidence of gas in the soft tissue, indicative of advanced disease and a marker of worse prognosis. The presence of a thickened fascia can make it difficult to differentiate the underlying structures. Yet, there is always the possibility of comparing with another similar unaffected structure, usually the other limb. Ultrasound can also be helpful to guide fluid drainage if a collection is present and rule out deep vein thrombosis.

Contrary to plain radiographs and CT, ultrasound has no ionizing radiation and is also relatively cheap. These features allow the clinician to repeatedly monitor the evolution of the patient, searching for possible local or systemic complications.

**Fig. 5:**
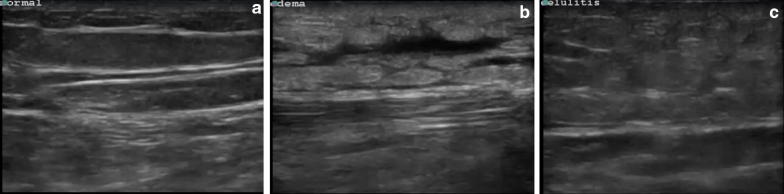
Point-of-care ultrasound scan of a different patient showing normal soft tissue (**a**), peripheral edema (**b**) and cellulitis (**c**) using a 15–6 MHz linear probe

Distinguishing NSTI from cellulitis (Fig. 5), the main alternative diagnosis, or other causes of soft-tissue edema using POCUS is not always possible, especially if it involves the deeper fascia, as a thickened fascia can appear in both cases [19]. Some ultrasound findings, including an irregularity of the fascia and an abnormal fluid collection along the fascial plane can help distinguish between the NSFI and cellulitis [16]. However, this evaluation is more difficult, strengthening the importance of taking the information obtained by POCUS together with the clinical evaluation and laboratory results.

Evaluation of deep structures can be difficult because of the hyperechogenic inflamed subcutaneous tissue that reflects most of the ultrasound beams. Also, as with other applications of POCUS, it is a user-dependent technique that requires some practice before being correctly used.

CT and MRI are still the most sensitive methods of diagnosis. CT is the most used imaging method to make the diagnosis, with a higher spacial resolution than ultrasound that allows the evaluation of deeper structures not accessible to ultrasound. It has a sensitivity of 80% in detecting NSTI [20]. MRI is the gold standard for the non-invasive diagnosis of NSTI, with sensitivity and specificity varying according the used criteria (presence of gas has a 100% specificity while extensive involvement of the intermuscular fasciae has a 100% sensitivity) [21]. However, these exams are less available, more time-consuming and expensive. The patient must also be transferred to the radiology department, which can lead to delays in the diagnosis and initiation of the therapy and lack of appropriate monitoring.

In this case, combining a high suggestive clinical evaluation and the documentation of the traditional signs observed in NSTI by POCUS, including the presence of gas in the tissue, allowed for a faster initiation of appropriate therapy and prompt surgical debridement, without having to transport the patient to a time-consuming exam like CT or MRI that could otherwise delay the diagnosis and the treatment. This case also shows complete remission of the ultrasound signs 1 month after the event.

## Conclusion

This case shows the utility of bedside ultrasound for the decision-making process in a disease where an early diagnosis is important. This information should always be used together with the clinical judgement, as it has a low sensitivity.

Further studies are necessary to understand the implications of this exam in the treatment and prognosis of this disease and if a protocolized approach would be beneficial in this population.

## Data Availability

Data sharing is not applicable to this article as no datasets were generated or analyzed during the current study.

## Abbreviations

CT: computerized tomography
MRI: magnetic resonance imaging
NSTI: necrotizing soft tissue infections
POCUS: point-of-care ultrasound
